# Facile Synthesis of Cu-Doped TiO_2_ Particles for Accelerated Visible Light-Driven Antiviral and Antibacterial Inactivation

**DOI:** 10.1021/acsaenm.4c00176

**Published:** 2024-05-03

**Authors:** Zachary S. Campbell, C. Roland Ghareeb, Steven Baro, Jacob Mauthe, Gail McColgan, Aram Amassian, Frank Scholle, Reza Ghiladi, Milad Abolhasani, Elizabeth C. Dickey

**Affiliations:** † Department of Chemical and Biomolecular Engineering, North Carolina State University, 911 Partners Way, Raleigh, North Carolina 27603, United States; ‡ Department of Materials Science and Engineering, North Carolina State University, 911 Partners Way, Raleigh, North Carolina 27603, United States; § Department of Chemistry, North Carolina State University, 2620 Yarbrough Drive, Raleigh, North Carolina 27695-8204, United States; ∥ Department of Biological Sciences, North Carolina State University, 3510 Thomas Hall, Campus Box 7614, Raleigh, North Carolina 27695, United States; ⊥ Department of Materials Science and Engineering, Carnegie Mellon University, 5000 Forbes Ave, Pittsburgh, Pennsylvania 15213, United States

**Keywords:** doping, TiO_2_, antimicrobial photodynamic inactivation, photosensitizer, antimicrobial coating

## Abstract

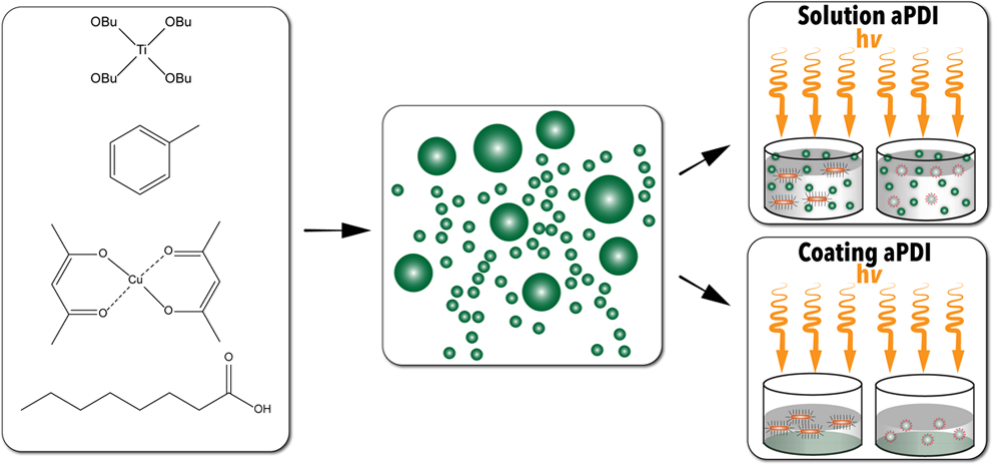

In this work, we present a facile and scalable hydrolysis-based route for the synthesis of copper-doped TiO_2_ particles for highly effective light-activated antiviral and antibacterial applications. The performance of the synthesized Cu-doped TiO_2_ particles is then evaluated using solution-phase antimicrobial photodynamic inactivation assays. We demonstrate that the Cu-doped TiO_2_ particles can successfully inactivate a wide range of pathogens with exposure to light for 90 min, including bacteria ranging from methicillin-resistant *Staphylococcus aureus* (99.9999%, ∼6 log units) to *Klebsiella pneumoniae* (99.93%, ∼3.3 log units), and viruses including feline calicivirus (99.94%, ∼3.4 log units) and HCoV-229E (99.996%, ∼4.6 log units), with the particles demonstrating excellent robustness toward photobleaching. Furthermore, a spray-coated polymer film, loaded with the synthesized Cu-doped TiO_2_ particles achieves inactivation of methicillin-resistant *S. aureus* up to 99.998% (∼4.8 log units). The presented results provide a clear advance forward in the use of metal-doped TiO_2_ for aPDI applications, including the scalable synthesis (kg/day) of well-characterized and robust particles, their facile incorporation into a nontoxic, photostable coating that may be easily and cheaply applied to a multitude of surfaces, and a broad efficacy against drug-resistant Gram-positive and Gram-negative bacteria, as well as against enveloped and nonenveloped viruses.

## Introduction

The SARS-CoV-2 virus responsible for the COVID-19 pandemic caused substantial damage to the global economy, claimed many lives, and has had a prominent effect on the social landscape. At present, the virus has claimed over 6.5 million lives globally, with more than 1,000,000 deaths in the U.S. alone.^1^ Transmitted primarily via aerosolized droplets, SARS-CoV-2 has demonstrated the ability to survive on a wide variety of surfaces for extended periods of time, lasting as long as 7 days.^2,3^ While the recent vaccines have shown tremendous success at preventing the spread of the virus, the Delta and newer Omicron variants exhibit significantly higher infectivity and transmission, even in fully vaccinated individuals.^4^ Beyond COVID-19, hospital-acquired infections (HAIs) represent another rapidly growing health concern. Prior to the COVID era, HAIs affected 1 out of every 20 hospitalized patients, resulting in 100,000 deaths in the U.S. annually.^5^ The spread of HAIs can be linked to contaminated surfaces, which may have been improperly cleaned or readily recontaminated with pathogens by healthcare workers or patients.^5^ Pathogens also persist commonly on surfaces that are not routinely cleaned, including hospital curtains and linens, with *Acinetobacter baumannii* and *Staphylococcus aureus* surviving weeks to months on contaminated materials.^7,8^ The role of these healthcare industry textiles as an infection route illustrates the importance of self-sterilizing materials to reduce the spread of HAIs.^9−12^

The need for long-lasting passively antimicrobial materials for preventing the spread of both the COVID-19 virus and HAIs has sparked rapid investigations into the fabrication of antimicrobial surfaces and textiles.^13−15^ There have been a wide variety of approaches to address this pressing issue ranging from copper (Cu) coatings to polymers capable of drastic pH change.^16−22^ While existing methods can be effective at preventing surface-based transmission, many are difficult to effectively apply to surfaces or suffer drawbacks such as toxic leachates.^23,24^ One particularly interesting method to circumvent these problems involves incorporation of visible light photosensitizers (PS) into materials for antimicrobial photodynamic inactivation (aPDI). These techniques are especially appealing due to the availability of photons from solar irradiation and ambient fluorescent light sources in hospitals. Photodynamic inactivation is a branch of photomedicine that utilizes a PS, oxygen, and light to produce a range of reactive oxygen species (ROS).^25^ These ROS are generated either through electron transfer reactions (Type I), which produce hydroxyl radicals (OH), superoxide (O_2_^–^), and peroxides (H_2_O_2_), or through energy transfer reactions (Type II), which produce singlet oxygen (^1^O_2_).^25−30^ The oxidative damage caused by ROS to inactivate pathogens is advantageous as bacteria and viruses are less likely to develop resistance, with no known resistance mechanism to singlet oxygen.^25^ A wide variety of PS, ranging from organic dyes to naturally occurring tetrapyrroles, have been successfully incorporated into materials through different methods to grant antimicrobial efficacy against both bacteria and viruses.^31−34^ Unfortunately, many organic PS possess low thermal stability and suffer from photobleaching,^35^ making them difficult to adapt to industry and driving the search for other PS that can be used as antimicrobial agents.

Numerous materials, including metal oxides, mixed metal oxides, chalcogenides, and III–V semiconductors, have been widely explored for photoinduced applications ranging from photovoltaics to photomedicine.^36−38^ Among these materials, titanium dioxide (TiO_2_) has been extensively studied, as it is known to be nontoxic, readily available, affordable, and possesses excellent chemical, physical, and thermal stability. Most importantly, various TiO_2_-based materials have shown considerable efficacy in degradation of organics and dyes, antimicrobial properties, and overall ROS production.^39−42^ Additionally, TiO_2_ has also been used in water and wastewater treatment,^43−45^ air purification,^46,47^ energy production,^48^ pigments, and light-driven organic transformations.^49^

The overall photocatalytic activity of TiO_2_ is attributed to its unique and tunable physicochemical properties, including particle size, morphology, crystal structure, defect types, defect concentration, and composition. These properties cumulatively affect the band structure and optical properties of TiO_2_, and ultimately dictate the photocatalytic activity. TiO_2_ possesses a wide band gap with two primary polymorphs, anatase and rutile, having 3.2 and 3.0 eV band gaps, respectively. Despite the larger band gap, which requires more energetic photons, further beyond the visible spectrum, to excite electrons from the valence band to the conduction band, anatase is widely regarded as the superior photocatalytic phase. Regardless of the phase, the band gap (3.0–3.2 eV) of TiO_2_ places its absorption peak in the near-ultraviolet (UV) region, a significant obstacle for using TiO_2_ effectively in applications with facile access to visible light. This has been addressed in part by the implementation of hybrid organic–inorganic photosensitizers combining TiO_2_ with various porphyrins to improve light absorption and antimicrobial inactivation.^50,51^ However, these materials still likely suffer from some of the main drawbacks associated with organic PS, namely photobleaching. Other methods, particularly transition-metal doping, have demonstrated promising results toward improving visible light absorbance and enhancement of photocatalytic activity in TiO_2_-based materials for contaminant oxidation,^52^ CO_2_ reduction,^53^ nitrogen fixation,^54^ and others. Briefly, the addition of transition-metal dopants is a method of introducing defects and/or midgap states which alter the light wavelengths that may be absorbed, thus allowing other, lower-energy photons to be absorbed by the material and excite electrons for the generation of ROS.

Copper is among the most commonly explored dopant metals, and a number of studies have produced various iterations of both Cu-doped TiO_2_ and different Cu-TiO_2_ composite materials for antimicrobial applications. A variety of synthetic techniques, including sol–gel,^55,56^ high pressure/temperature solvothermal,^57,58^ chemical vapor deposition,^59^ and high-energy ball milling,^60^ have been utilized to produce Cu-doped TiO_2_, which has been tested against model pathogens (i.e., *Escherichia coli* and *S. aureus*) for photoinduced antimicrobial inactivation. For example, Mathew et al.^56^ and Yadav et al.^55^ demonstrated the synthesis of Cu-doped TiO_2_ particles using various sol–gel methods which were then utilized for antimicrobial inactivation upon exposure to visible light. The materials produced by Mathew et al. were able to inactivate both *E. coli* and *S. aureus* between 5 and 6 log units in 30 min using a solar simulator. Meanwhile, Yadav et al. were able to more modestly inactivate 90% of *S. aureus* (1 log unit) and ∼45% of *E. coli* (0.5 log unit) in 90 min upon irradiation by a series of fluorescent lamps. Foster et al.^59^ demonstrated the preparation of Cu-TiO_2_ and Ag-TiO_2_ films deposited via chemical vapor deposition, which were utilized to inactivate *E. coli* and *S. aureus* in both dark and light conditions. The Cu-TiO_2_ films were demonstrated to inactivate *E. coli* up to 6 log units under UVA irradiation for 2 h. However, substantial inactivation was also observed in dark conditions, which likely indicates some degree of cytotoxicity due to copper leaching. Moreover, the aforementioned studies did not evaluate the antiviral activity of the materials, nor were their efficacy examined against drug-resistant pathogens, such as the ESKAPE pathogens responsible for many of the most difficult-to-treat nosocomial infections.^61^ Therefore, it is desirable to develop a facile, scalable synthetic technique for the preparation of well-characterized visible light-absorbing microparticles that have been demonstrated to be effective against a variety of viruses (i.e., enveloped, nonenveloped) and bacteria (i.e., Gram-positive, Gram-negative), including drug-resistant strains. Furthermore, it is necessary to demonstrate the resilience of these particles toward photobleaching, as well as the incorporation of these materials into a robust coating that largely retains the desired visible light-driven antimicrobial inactivation without leaching harmful ions.

In response, we report a facile, scalable hydrolysis-based synthesis of Cu-modified TiO_2_ powders and their effectiveness against a wide range of pathogens, including both bacteria and viruses. The synthesis and biocidal activity of Cu-doped TiO_2_ powders possessing Cu loadings ranging from 3 mol % to 10 mol % Cu: mol Ti are reported in this study. The materials were synthesized by a one-pot hydrolysis-based synthetic technique with high yield (approaching 100%), relatively low cost, and high throughput (approaching kg/day scales). The synthesized particles were capable of up to 6 log units of inactivation against methicillin-resistant*S. aureus* (MRSA) in solution-phase antimicrobial photodynamic inactivation tests upon irradiation for 90 min by a photodynamic therapy (PDT) lamp with a 400–700 nm output, and their robustness toward photobleaching was assessed under extended illumination conditions. Selected materials were then tested against a range of other model pathogens, including Gram-positive bacteria (i.e., vancomycin-resistant *E. aureus*), Gram-negative bacteria (i.e., multidrug-resistant *A. baumannii* and *Klebsiella pneumoniae*), a nonenveloped virus (i.e., feline calicivirus), and an enveloped virus (i.e., HCoV-229E), with these latter two representing a more novel evaluation of Cu-doped TiO_2_ for antiviral applications. The synthesized Cu-doped TiO_2_ particles were then applied to filter paper through spray coating and exhibited 99.998% (4.8 log units) inactivation against MRSA with no observed photobleaching, demonstrating potential for immediate application for passive self-cleaning surfaces and personal protective equipment.

## Methods

### Materials

Titanium(IV) *n*-butoxide (99+%, Alfa Aesar) and acetone (ACS reagent, Alfa Aesar) were purchased from Fisher Scientific. Copper(II) acetylacetonate (97%) and octanoic acid (≥98%) were purchased from Sigma-Aldrich. The photo-cross-linkable SbQ-PVA polymer with 4.1 mol % functional SbQ groups was supplied by Polysciences, Inc. Deionized water (18.2 MΩ cm) and dry ice were sourced internally. YSZ milling media (3 mm, McMaster Carr) was used for powder milling, and BYK 156 (BYK) was used as the milling dispersant. All materials were used as received. Buffer salts for the preparation of phosphate-buffered saline (PBS) solution and ultrapure nitric acid for inductively coupled plasma-optical emission spectroscopy (ICP-OES) analysis were purchased from Fisher Scientific. Tryptic soy broth was purchased from Teknova, and all media and buffer solutions were prepared in ultrapure water provided by an Easypure II system (Barnstead).

### Synthesis of Cu-TiO_2_

Cu-doped TiO_2_ particles were prepared by mixing toluene, titanium(IV) *n*-butoxide, and octanoic acid at a volume ratio of 6.5:2.5:1 with nominal copper(II) acetylacetonate loadings from 3 to 10 mol % Cu: mol Ti. These compositions were selected due to inadequate modulation of visible light absorbance at lower Cu loadings (i.e., 1, 2%) (Supporting Information(SI)). The mixture was then sonicated to ensure a homogeneous solution (∼10 min). Acetone (20 mL) was then added to the mixture under sonication, sonicating for approximately 1 min, after which 10 mL of deionized water was rapidly injected to hydrolyze the titanium(IV) *n*-butoxide, causing the formation of green precipitates. The precipitates were separated (Whatman P2 filter paper) and dried at ambient temperature. The resulting materials were annealed at temperatures ranging from 400 to 600 °C in a muffle furnace for 1 h with a 5 °C/min ramp rate.

### Instrumentation and Characterization

Crystal structure was characterized using a PANalytical Empyrean X-ray diffractometer, and the phases present in scans were identified with HighScore Plus and refined in GSAS II software packages. Scanning electron microscopy (SEM) and energy dispersive X-ray spectroscopy (EDS) (FEI Verios 460L), were used to identify particle morphology and composition and to evaluate the particle size distribution. Individual particle diameters were measured digitally from high-resolution SEM images using ImageJ. Transmission electron microscopy (TEM) (FEI Titan) and electron diffraction were also used to evaluate the presence and distribution of copper in the synthesized materials. Diffuse reflectance ultraviolet–visible (UV–vis) spectroscopy was used to measure the absorption spectra of the materials and the effective change in band gap. Differential thermal analysis and thermogravimetric analysis (DTA/TGA) were used to identify exothermic reactions and mass loss of the product during heat treatment. X-ray photoelectron spectroscopy (XPS) (SPECS) was used to determine the local bonding environment and valence of the transition metals in the synthesized particles. Inductively coupled plasma-optical emission spectroscopy (ICP-OES) measurements were utilized to investigate the possible leaching of Cu ions into solution, as well as the total amount of incorporated Cu.

### Material Coating

The coating protocol was adapted from previous publications.^32,34^ Briefly, *N*-methyl-4(4′-formyl-styryl)pyridinium methosulfate acetal poly(vinyl alcohol) (SbQ-PVA) was dissolved in deionized (DI) water at a concentration of 10% w/v (SbQ-PVA/water) and stirred until fully dissolved. The as-synthesized Cu-TiO_2_ materials were prepared via ball milling for 12 h using BYK 156 as a dispersant and dried by lyophilization in a Labconco freeze-dryer. The powders were subsequently added to the SbQ-PVA solution at a loading level of 10 mg/mL and sonicated for 1 h prior to coating. Whatman filter paper, a common model substrate for fibrous materials (e.g., cellulose, cotton) found in a hospital setting (e.g., hospital bed linens, examination room paper),^62,63^ was cut into squares measuring 8 cm × 8 cm, with one side of each spray-coated until saturated (1 mL) using a Master Airbrush Model G22 with a 0.3 mm fluid tip. Following coating, the material was cured in a MelodySusie UV light (36 W, 365 nm) for 30 min. A secondary “sealant” coat consisting of a PS-free aqueous SbQ-PVA solution was then applied (1 mL), followed by a final UV cure for an additional 30 min. Control samples were prepared by spray coating filter paper with only PS-free SbQ-PVA solution. BYK 156 was also tested individually for antimicrobial activity and showed no lethality in coatings or solution without Cu-TiO_2_ particles present. Once cured, the coated filter paper(s) was gently washed in ultrapure water overnight to remove any excess/unbound polymer or PS. Prior to antimicrobial testing, the coated samples were cut with a custom hole punch into circles measuring either ∼0.5 or 1 cm in diameter.

## Antimicrobial Studies

### Bacteria Culture

Antibacterial photodynamic inactivation assays were performed with Gram-positive methicillin-resistant *S. aureus* (MRSA, ATCC-44) and Gram-negative multidrug-resistant *A. baumannii* (MDRAB, ATCC-1605). Cultures were grown in 5 mL of tryptic soy broth (TSB) and LB-Miller broth containing 5 mg/mL tetracycline HCl for MRSA and MDRAB, respectively. *K. pneumoniae* (ATCC BAA-2146; NDM-1 positive multidrug-resistant variant) was grown in BD Difco Nutrient Broth #234000 with 100 μg/mL ampicillin. Vancomycin-resistant*Enterococcus faecium* (ATCC-2320) was grown in BD Difco Bacto Brain Heart Infusion 237500 with 100 μg/mL ampicillin. Bacteria cultures were incubated at 37 °C in an orbital shaker operated at 250 rpm until a concentration of 1–4 × 10^8^ colony-forming units per mL (CFU/mL) was reached; the concentration was determined via optical density measurements at 600 nm (OD_600_) using a Thermo Electron Corporation Genesys UV–vis scanning spectrophotometer. The culture was centrifuged for 5 min (3374*g*) to obtain the pellet, the supernatant was discarded, and the culture was resuspended in 5 mL of phosphate-buffered saline (PBS; aqueous solution of 170 mM NaCl, 3.4 mM KCl, 10.0 mM Na_2_HPO_4_, 1.8 mM KH_2_PO_4_, pH 7.2) containing 0.05% Tween-80.

### Antimicrobial Photodynamic Inactivation (aPDI) in Solution

Bacterial inactivation studies were performed in sterile 24-cell well plates (BD-Falcon). Synthesized Cu-TiO_2_ powders were suspended in sterilized PBS solution and sonicated 60 min prior to use. Aliquots of 200 μL bacterial solution and 200 μL PS solution (10 mg/mL) were combined in each well. The plate was wrapped in plastic wrap and wells were subsequently illuminated for 30, 60, and 90 min at either 65 ± 5 or 85 ± 5 mW/cm^2^ using a LumaCare LC-122 incoherent visible light source equipped with an OSRAM 64653 HLX Xenophot bulb (250 W, 24 V) and employing a LUM V fiber optic probe (400–700 nm band-pass filter) with ±3% average transmittance. Lamp irradiance was confirmed prior to each experiment using an Ophir Orion power meter. All illumination studies were performed in triplicate, with two dark controls and one PS-Free (light) control to determine final growth concentration (in CFU/mL). Dark controls consisted of cell well plates containing solutions identical to the standard aPDI experiments covered in aluminum foil to completely eliminate light exposure.

After illumination was complete, 40 μL aliquots were withdrawn from each of the wells and added to a 360 μL PBS aliquot to serve as a 1:10 dilution. This procedure was repeated 5 times to generate six 10-fold serial dilutions for each well. A 10 μL aliquot from each dilution was pipetted onto six-column-gridded square plates that were previously prepared with antibiotic-free TSB/agar or LB-Miller broth/agar for MRSA and MDRAB respectively. Plates were incubated in dark conditions (i.e., covered in aluminum foil) overnight at 37 °C. Separation of the particles prior to incubation was unnecessary due to the use of dark incubation conditions and comparison with dark control experiments. Colony-forming units were counted and the corresponding level of bacterial inactivation was calculated by dividing the CFU/mL count of the illuminated/dark samples by the corresponding PS-free control. Statistical significance was assessed via a two-tailed, unpaired Student’s *t* test. Results were considered to be significant when the *p*-value was <0.05.

### Repeated Illumination Experiments

Experiments were conducted to determine the degree to which the synthesized particles could be photobleached to provide a comparison to well-known organic PS. In these tests, synthesized particles were stirred in solution under light exposure using the equipment mentioned previously (85 ± 5 mW/cm^2^, 400–700 nm, 90 min) for a total of 4 illumination periods. Between each illumination period, the powder was centrifuged, the supernatant was discarded, and the powder was resuspended in PBS. After 4 cycles of irradiance by the PDT lamp, the particles were then exposed to a bacterial solution containing MRSA and irradiated to determine the antimicrobial efficacy at 450 min of illumination.

### aPDI on Coated Materials

Coated materials were cut into disks (∼1 cm diameter) and fitted into the well bottoms of a 24-well plate (3 PS-containing samples, 1 PS-free control sample). A resuspended bacterial solution (200 μL) was added and uniformly deposited on top of each sample. An identical plate protected from light with aluminum foil was prepared for the purpose of dark control. Illumination and serial dilutions were performed in an identical manner to the solution-based antimicrobial assays.

### Antiviral Photodynamic Inactivation in Solution

Viral inactivation studies were performed in 96-well plates. Briefly, 100 μL of suspended Cu-TiO_2_ powders was combined with 75 μL of modified Eagle’s medium supplemented with 1% FBS, 1% antibiotics 1% HEPES buffer, and 25 μL of virus solution. Wells were illuminated for 90 min with 65 ± 5 mW/cm^2^ using a LumaCare LC-122 incoherent visible light source equipped with an OSRAM 64653 HLX Xenophot bulb (250 W, 24 V) and employing a LUM V fiber optic probe (400–700 nm band-pass filter) with 95 ± 3% average transmittance. Controls included virus only (no Cu-TiO_2_) and dark controls for each powder. Samples were transferred into Eppendorf tubes and centrifuged for 2 min (3700*g*) prior to removal of supernatants. Virus infectivity of supernatants was determined by TCID_50_ titration on a cell line suitable for each virus.

HCoV-229E was grown and titered on the human hepatoma cell line Huh-7 and feline calicivirus (FCV) was grown and titered on Crandall-Reese feline kidney cells. TCID_50_ assay plates were incubated at 37 °C in a 5% CO_2_ atmosphere for 96, and 48 h for HCoV-229E and FCV, respectively. Cytopathic effect was determined by light microscopic observation, and TCID_50_ titers were calculated using the Spearman–Karber method.^64^

## Results and Discussion

### Materials Characterization

**Figure 1:**
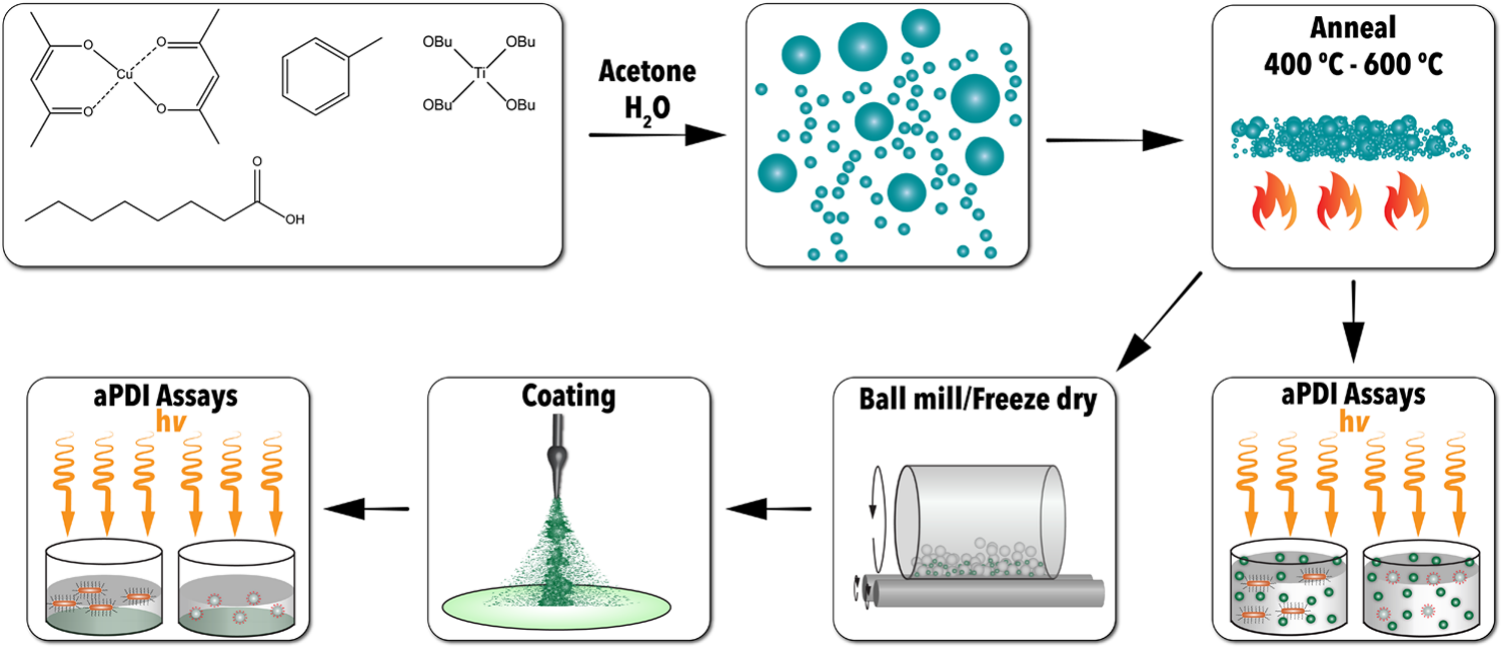
Procedure utilized for the preparation of Cu-doped TiO_2_ particles and coatings, as well as subsequent use in antimicrobial photodynamic inactivation assays.

Figure 1 overviews the procedure by which materials were synthesized, characterized, and tested for biocidal applications. Synthesized materials were characterized while being screened via solution-based aPDI assays. The Cu-TiO_2_ coatings were further characterized and implemented for aPDI assays to evaluate their feasibility for passive self-cleaning coatings to minimize microbial transmission. As will be discussed below, the 8 mol % Cu powders annealed at 500 °C (denoted 8%–500 °C) and 600 °C (8%–600 °C) were found to generally exhibit the best aPDI efficacy, and results from those samples will therefore be highlighted in the discussion and primarily compared with the 3%–400 °C annealed sample. Lower Cu loadings (i.e., 1, 2%) were found to insufficiently alter the visible light absorbance of the synthesized materials, and thus were not tested.

**Figure 2:**
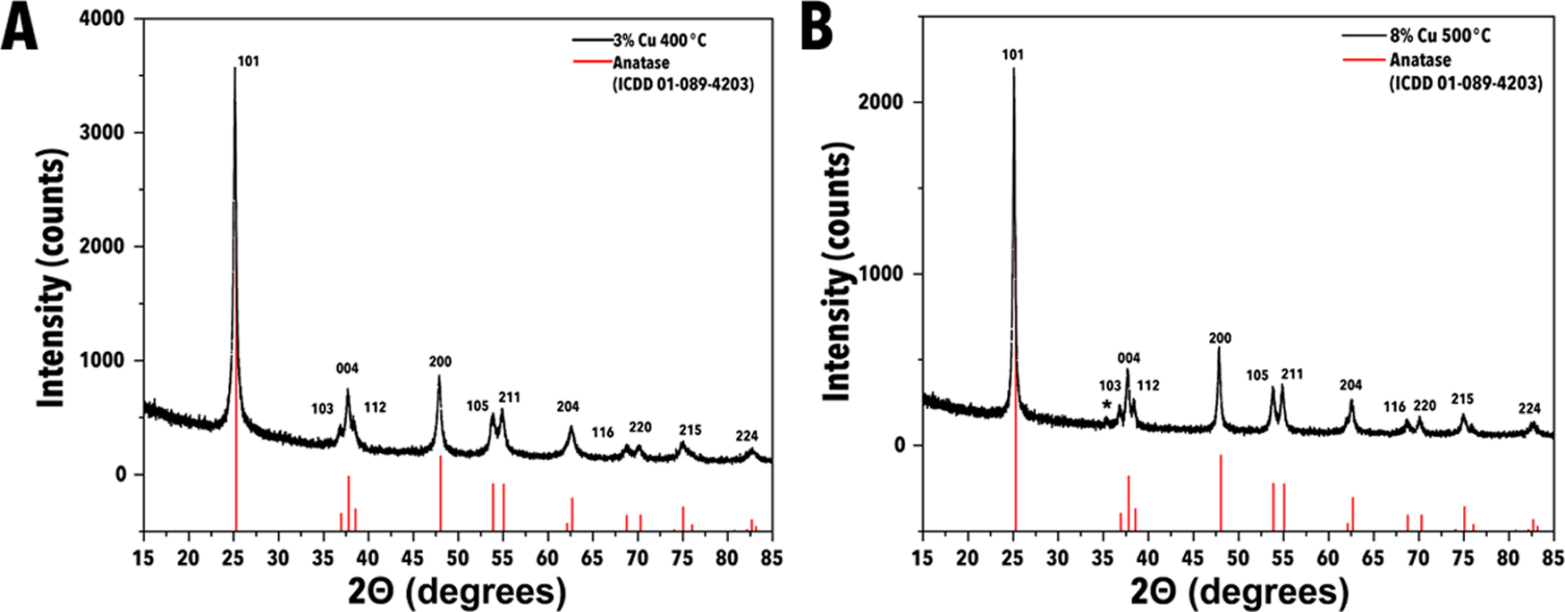
XRD spectra of synthesized particles. (A) XRD spectrum of the 3% Cu-doped TiO_2_ annealed at 400 °C (3%–400 °C) particles. (B) XRD spectrum of the 8% Cu-doped TiO_2_ annealed at 500 °C (8%–500 °C) particles. A minor peak denoted with * is consistent with the hkl reflection of CuO.

The crystal structures of the synthesized powders after heat treatment were measured by X-ray diffraction (XRD), and the phases were identified by comparing peak positions and relative intensities to ICDD standards. The results for 3% Cu and 8% Cu loadings, annealed at 400 and 500 °C, respectively, are shown in Figure 2, while the remaining XRD spectra may be found in Figure S3. In all samples annealed at 400 or 500 °C, the XRD spectra show the anatase TiO_2_ structure, and no other crystalline phases are observed within the detectable limits of XRD. Upon heating to 600 °C, the formation of both CuO and rutile TiO_2_ was observed in both the 8% and 10% Cu-doped samples. All samples exhibited a leftward shift in the measured spectra, corresponding to a change in their lattice parameters and an increase in strain relative to anatase TiO_2_. To further evaluate Cu doping in the TiO_2_ lattice, the samples annealed at 500 °C were compared to a control sample synthesized using the same method but without any Cu (denoted 0%–500 °C). The *a* = *b* and *c* lattice parameters evaluated by Rietveld refinement are presented in Table 1.

**Table 1:** Lattice Parameters Evaluated via Rietveld Refinement for Samples Annealed at 500 °C

sample	*a* = *b* (Å)	*c* (Å)
0%–500 °C	3.787	9.495
3%–500 °C	3.790	9.512
8%–500 °C	3.792	9.507
10%–500 °C	3.793	9.509

As seen in Table 1, the Reitveld refinement of the 0%–500 °C control resulted in lattice parameters of *a* = *b* = 3.787, and *c* = 9.495 Å (standard anatase TiO_2_: *a* = *b* = 3.784 Å, *c* = 9.515 Å), which is indicative of some degree of strain present in the lattice, likely attributable to the synthetic method utilized. When the Cu loading is increased, there is a corresponding shift in the lattice parameters, especially the *a* = *b* parameter, where a clear increasing trend is apparent, thus indicating Cu doping in the TiO_2_ lattice. While the *a* = *b* parameter increases as a function of Cu concentration, it only increases minimally when the Cu loading is increased from 8 to 10%, which likely indicates that the Cu has approached its maximum solubility in the TiO_2_ around 8% loading. This is further supported by the emergence of the CuO (hkl) reflection at 2θ = 35° in samples loaded 8% Cu or greater (see Figures 2 and S3).

**Figure 3:**
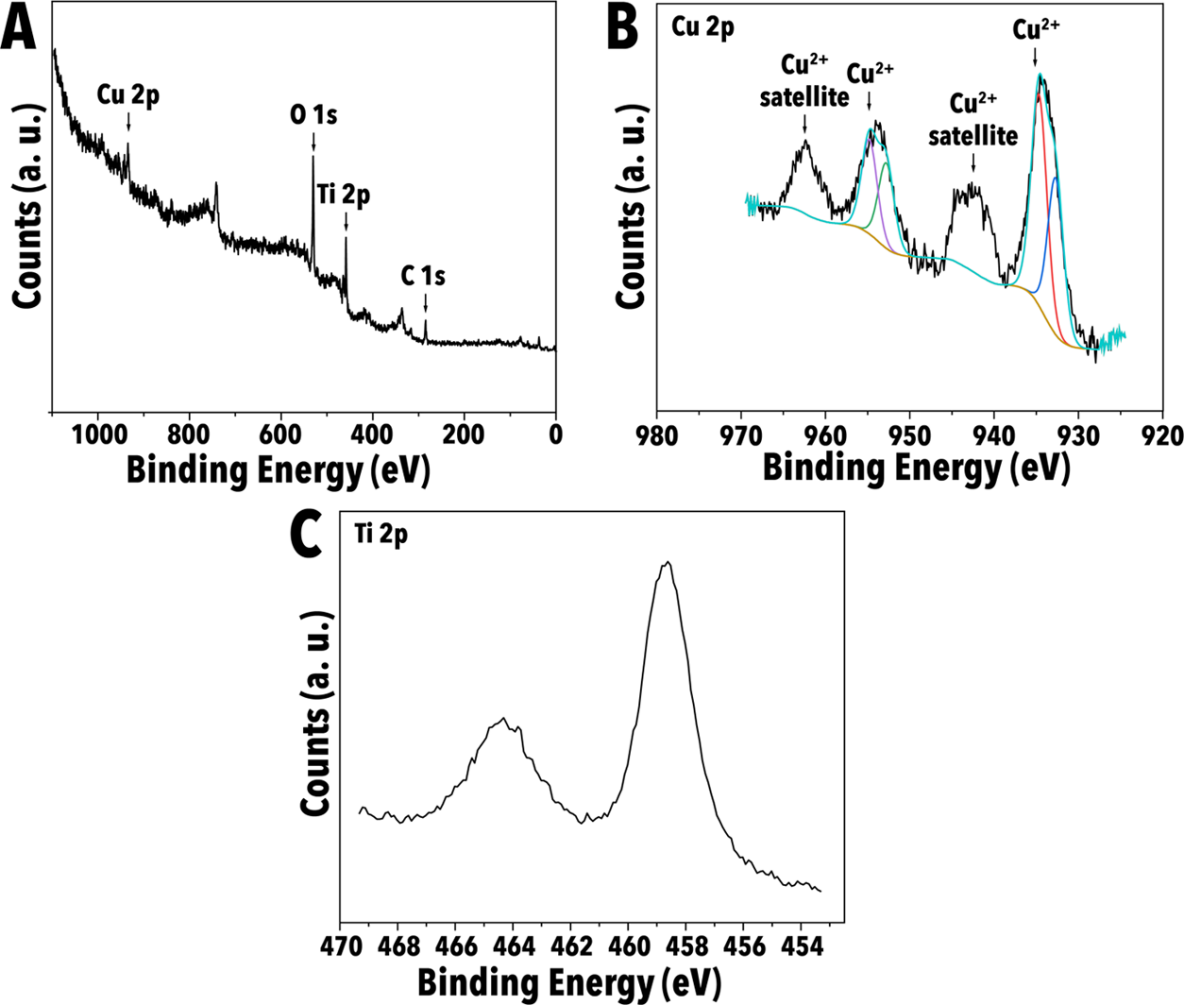
XPS scans of 8%–500 °C Cu-doped TiO_2_ particles. (A) Full XPS scan. (B) High-resolution Cu 2p scan. (C) High-resolution Ti 2p scan.

XPS measurements were performed to confirm the presence of copper in the TiO_2_ particles after thermal processing and to examine the local bonding environment of atoms near the surface. As expected, the full scan (Figure 3A) displayed peaks attributable to Cu, Ti, O, and C. Deconvolutions of the Cu 2p_3/2_ and 2p_1/2_ peaks in high-resolution XPS spectra indicated that multiple Cu oxidation states existed at the particle surface in all characterized materials, as evidenced by the peaks observed at 934.5/954.7 eV and 932.7/952.9 eV (Figure 3B). The Cu 2p peaks at 934.5 and 954.7 eV indicate the presence of Cu^2+^, whereas the shoulder observed in the deconvolutions near 932.7 and 952.9 eV indicate the presence of a more reduced copper species in the synthesized particles and may be attributable to either Cu–O–Ti bonds or the presence of Cu^1+^ species. Figure 3C presents the high-resolution Ti spectrum, which shows that the Ti exists predominantly as the expected Ti^4+^ oxidation state in TiO_2_ as indicated by the Ti 2p_1/2_ and Ti 2p_3/2_ peaks at 463.5 and 458.7 eV, respectively.

The concentration of Cu incorporated into the TiO_2_ particles was obtained using ICP-OES. The resulting Cu loading from nominal 3, 8, and 10% mole Cu:mole Ti compositions were found to have actual loadings of 2.75, 6.63, and 8.05%, respectively.

**Figure 4:**
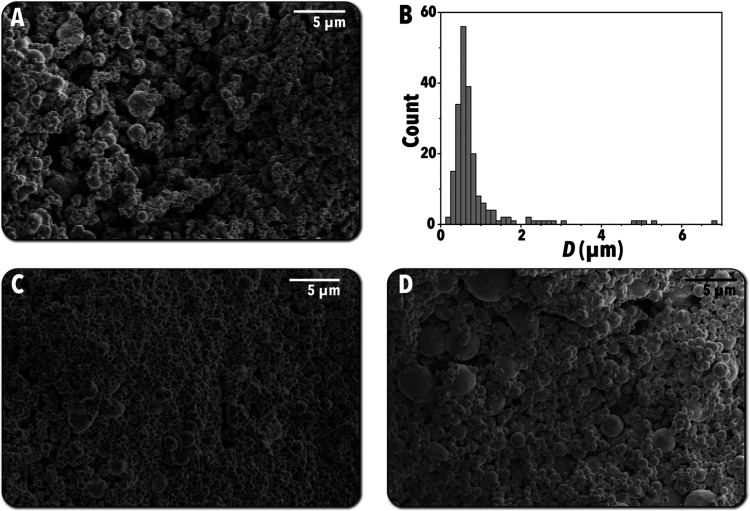
SEM data of synthesized particles. (A) SEM image of 8%–500 °C particles. (B) Measured size distribution of 8%–500 °C particles via SEM and ImageJ. (C) SEM image of 3%–400 °C particles. (D) SEM image of 8%–600 °C particles.

The particle sizes and morphologies were measured via SEM. Figure 4A shows a secondary electron SEM image of the 8%–500 °C particles, which shows a large number of small spherical particles of O(100 nm) with comparatively few outliers of larger sizes (O(1 μm)). Finally, a particle size distribution was measured from the SEM images (Figure 4A). Figure 4B shows the size distribution of the measured particles, where a substantial majority of the particles were observed to have diameters between 200 and 1000 nm, with a relatively small fraction possessing diameters between 1 and 2 μm as well as a handful of larger outliers. Similar particle sizes were observed in both the 3%–400 °C (Figure 4C) and 8%–600 °C (Figure 4D) samples.

**Figure 5:**
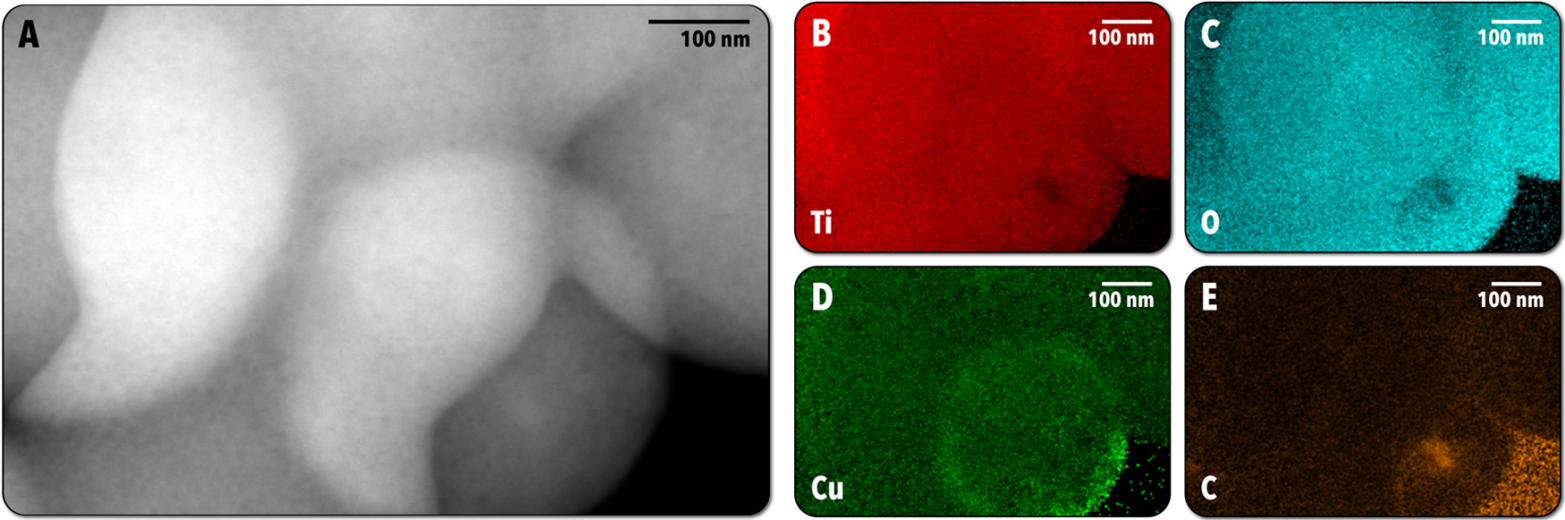
TEM-EDS data for 8%–500 °C particles. (A) High-angle annular dark-field  (HAADF) image of the particles. (B) Ti EDS map. (C) O EDS map. (D) Cu EDS map. (E) C EDS map.

The 8%–500 °C particles were also characterized using STEM-EDS to evaluate elemental distribution (Figure 5). Mapping was performed for Ti (Figure 5B), O (Figure 5C), Cu (Figure 5D), and C (Figure 5E). The particles are primarily composed of Ti and O, which are both clearly well distributed throughout the particles, with the exception of an apparent void in one of the imaged particles. Similarly, the Cu EDS map illustrates that the Cu is generally well mixed with the Ti and O, indicative of Cu doping in the TiO_2_ lattice. We note that one of the particles has surface-segregated copper, indicated by the higher concentration of green pixels at one of the particle surfaces in Figure 5D. However, this observation was uncommon, with the majority of particles possessing an even distribution of Cu. Finally, the C EDS map (Figure 5E) illustrates a low quantity of carbon distributed in the particles, indicating that most of the organic solvents and ligands are combusted or volatilized during drying and annealing steps. While there is apparently little carbon distributed in the particles, the void observed in the Ti and O EDS maps clearly corresponds to a substantial carbon inclusion, which indicates that the carbon may not always be fully combusted, resulting in some carbon-rich regions inside the particles, and perhaps leading to the inhomogeneous distribution of Cu in that particular particle. STEM-EDS data for the 3%–400 °C particles, in which Ti, O, and Cu were uniformly distributed throughout the particles, may be found in Figure S4. SEM-EDS spectra of the 8%–500 °C sample may be found in Figure S5.

**Figure 6:**
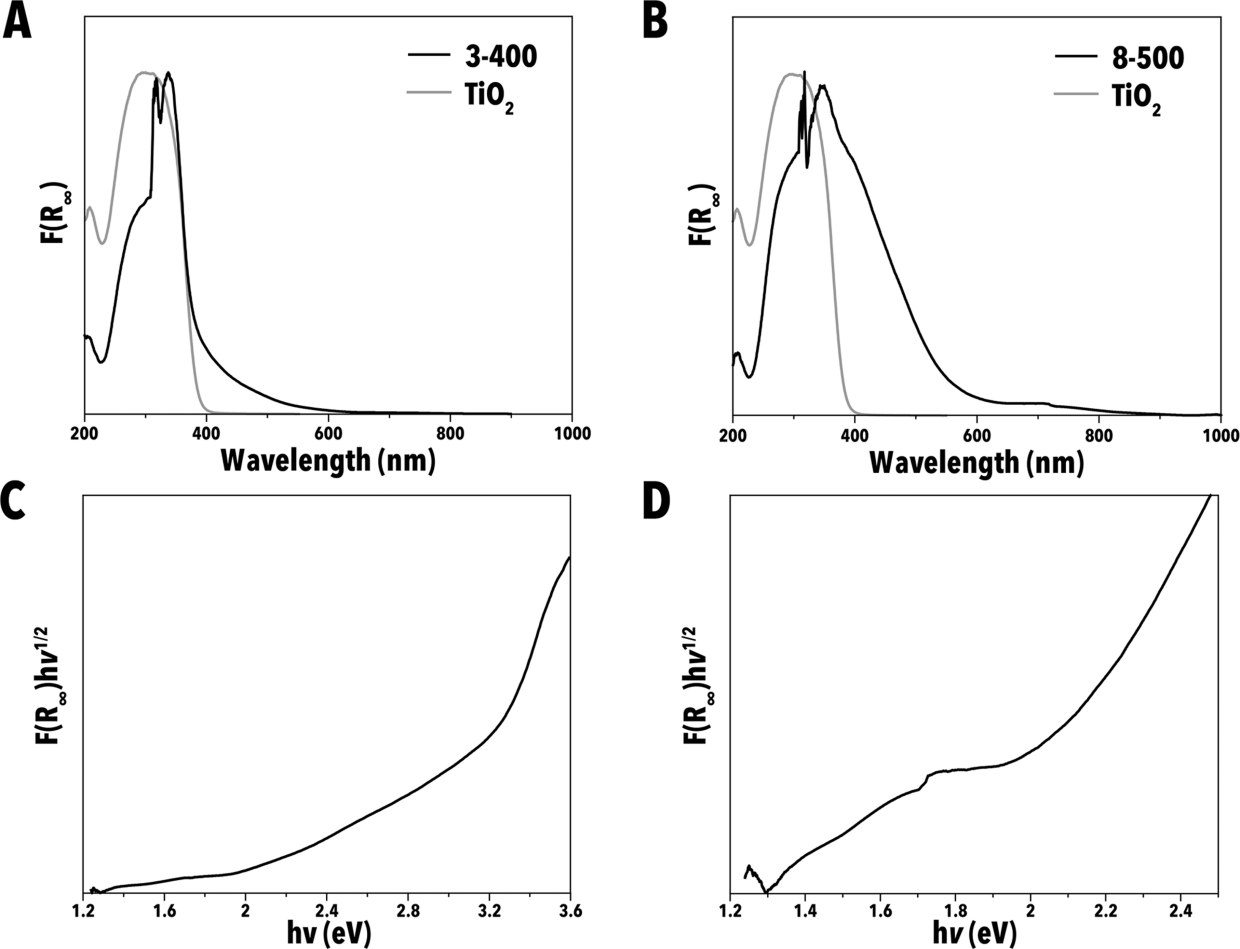
Diffuse reflectance UV–vis spectra and Tauc plots for 3%–400 °C and 8%–500 °C particles. (A) Kubelka–Munk transformed UV–vis spectrum of 3%–400 °C particles. The gray curve represents UV–vis spectrum of pure TiO_2_. (B) Kubelka–Munk transformed UV–vis spectrum of 8%–500 °C particles. The gray curve represents UV–vis spectrum of pure TiO_2_. (C) Tauc plot of the 3%–400 °C particles. (D) Tauc plot of the 8%–500 °C particles.

The 3%–400 °C and 8%–500 °C powders were analyzed using diffuse reflectance UV–vis spectroscopy. Figure 6A,B shows the Kubelka–Munk transformed UV–vis spectra of the 3%–400 °C and 8%–500 °C samples, respectively. Both exhibited increased absorption at wavelengths relative to anatase TiO_2_ at >400 nm due to Cu incorporation and the possible presence of additional defects from the soft synthetic method. This shift in absorbance is visualized in Figure 6A,B as the spectrum of anatase TiO_2_ is superimposed on the measured UV–vis spectra of the synthesized samples. As expected, a modest increase in absorbance was seen between 400 and 600 nm in the 3%–400 °C particles, while a much more substantial increase was observed in the 8%–500 °C sample, resulting in increased absorbance in the visible spectrum up to approximately 800 nm, while anatase TiO_2_ shows negligible absorbance beyond 400 nm. This increase in broad-band absorption in the visible spectrum is reflected in plot features known as Urbach tails, which are attributed to the introduction of midgap states. Figure 6C,D presents the absorbance in the Tauc plot formalism.

### aPDI Analysis Employing Cu-TiO_2_ Antibacterial Photodynamic Inactivation in Solution

Degussa P25 (**P25**) is a commercially available TiO_2_ powder commonly used in photocatalytic applications, including production of reactive oxygen species (ROS) like singlet oxygen (^1^O_2_). Unfortunately, due to its large band gap (3.2 eV) **P25** is only capable of being activated with higher energy UV radiation (<400 nm). When **P25** was tested as a potential photosensitizer (10 mg/mL) to be used in antimicrobial photodynamic inactivation via illumination for 90 min (85 ± 5 mW/cm^2^, 400–700 nm), it showed no bactericidal activity against methicillin-resistant *S. aureus* (MRSA; Figure S6).

The different Cu-doped TiO_2_ particles synthesized in the present study were tested for antimicrobial efficacy against MRSA (Figure S7). Initial studies were performed with lower particle concentrations, light intensities, and times (5 mg/mL, 65 ± 5 mW/cm^2^, and 30 min, respectively) in order to better distinguish which copper concentration and annealing temperature produced particles with the greatest antimicrobial efficacy. Of the particles tested, the particles doped with 3% Cu and annealed at 400 °C, as well as 8% Cu annealed at 500 and 600 °C (i.e., 3%–400 °C, 8%–500 °C, and 8%–600 °C, respectively) demonstrated the highest inactivations at 88, 93, and 99.5% inactivation, respectively. Due to their better performance, these particles were used for further antimicrobial studies.

**Figure 7:**
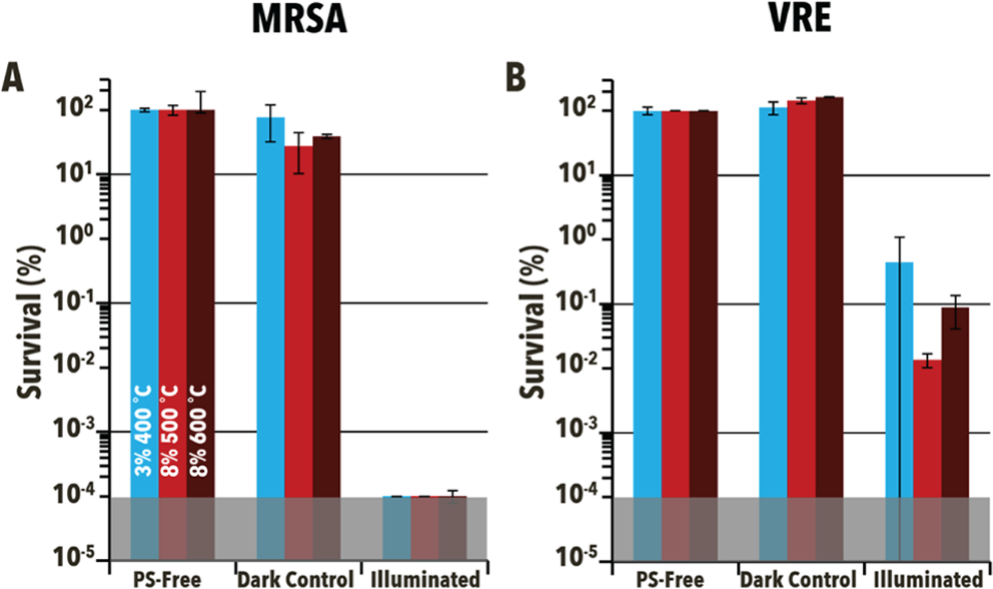
Solution-phase antibacterial photodynamic inactivation of (A) methicillin-resistant *Staphylococcus aureus* (MRSA) and (B) Vancomycin-resistant *Enterococci* (VRE) using 3%–400 °C, 8%–500 °C, and 8%–600 °C (blue, light red, and dark red, respectively). Both dark controls (Dark) and light reactions (Illuminated, illuminated for 90 min at 85 ± 5 mW/cm^2^, 400–700 nm) were at particle concentrations of 10 mg/mL and compared to photosensitizer-free controls (PSF) when determining percent survival. The shaded region represents the limit of detection. Error bars correspond to standard deviation.

The 3%–400 °C, 8%–500 °C, and 8%–600 °C Cu-doped TiO_2_ powders were further tested against MRSA at the higher particle concentration, light irradiance, and longer time (Figure 7). Each of the particles demonstrated inactivation to the limit of detection (99.9999%, 6 log units) of MRSA after the 90 min illumination period, an obvious improvement compared to **P25**. To further examine the particles’ efficacies against Gram-positive bacteria, antimicrobial studies were performed against vancomycin-resistant Enterococci (VRE) under the same conditions (Figure 7B). While not being able to inactivate VRE as well as MRSA, the particles still demonstrated the ability to disable VRE to at least 90%. 8%–500 °C inactivated VRE to the greatest extent (99.996%, ∼4.6 log units, *p* = 0.0001) with 8%–600 °C showing the second-best efficacy and 3%–400 °C demonstrating the worst (99.91%, ∼3.1 log units, *p* = 0.0001 and 99.5%, ∼2.5 log units, *p* = 0.0009, respectively).

**Figure 8:**
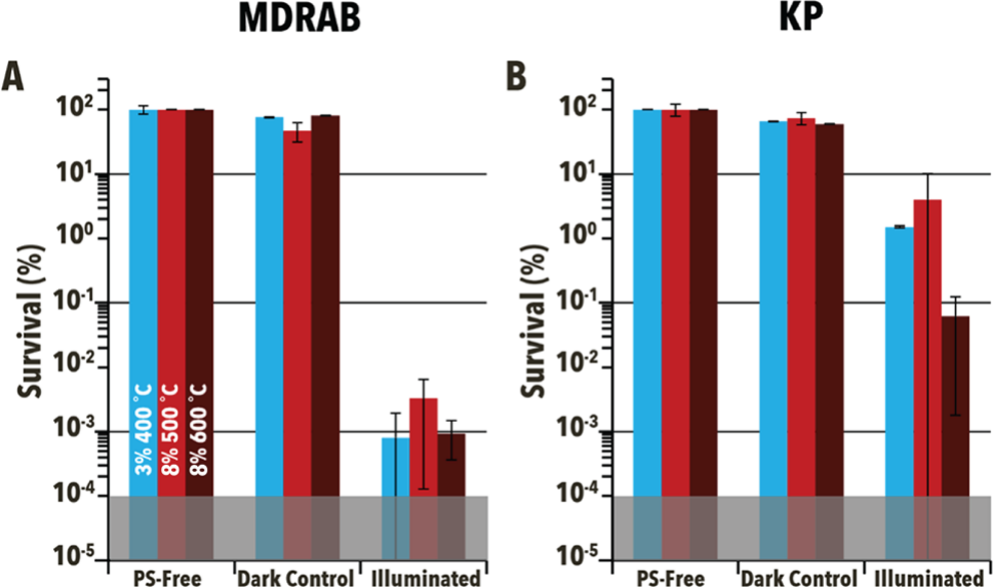
Experimental results obtained from solution-phase antibacterial photodynamic inactivation of (A) multidrug-resistant *A. baumannii* (MDRAB) and (**B**) *K. pneumonia* (KP) using 3%–400 °C, 8%–500 °C, and 8%–600 °C (blue, light red, and dark red, respectively). Both dark controls (Dark) and light reactions (Illuminated, illuminated for 90 min at 85 ± 5 mW/cm^2^) were at particle concentrations of 10 mg/mL and compared to photosensitizer-free controls (PSF) when determining percent survival. The shaded region represents the limit of detection. Experimental conditions are identical to Figure 7. Error bars correspond to standard deviation.

Further, aPDI assays utilizing 3%–400 °C, 8%–500 °C, and 8%–600 °C were performed against the Gram-negative bacteria multidrug-resistant *A. baumannii* (MDRAB) and *K. pneumoniae* (KP) (Figure 8). Under identical conditions as with Gram-positive bacteria, the particles showed less antimicrobial behavior overall, inactivating 99.9991% (∼5.1 log units, *p* = 0.001), 99.996% (∼4.6 log units, *p* = 0.0001), and 99.9990% (∼5 log units, *p* = 0.0001) against MDRAB and 98.5% (∼1.8 log units, *p* = 0.0001), 96% (∼1.6 log units, *p* = 0.0043), and 99.93% (∼3.3 log units, *p* = 0.0001) against KP for 3%–400 °C, 8%–500 °C, and 8%–600 °C, respectively.

While the antimicrobial efficacies were not as high for Gram-negative bacteria as they were for their Gram-positive counterparts, these results were not surprising. Gram-negative bacteria have been shown in previous studies to have a higher tolerance to ROS damage from aPDI.^26^ The tolerance is attributed to the double cell wall membrane possessed by Gram-negative bacteria, which adds further protection against radicals and other high-energy species.

**Figure 9:**
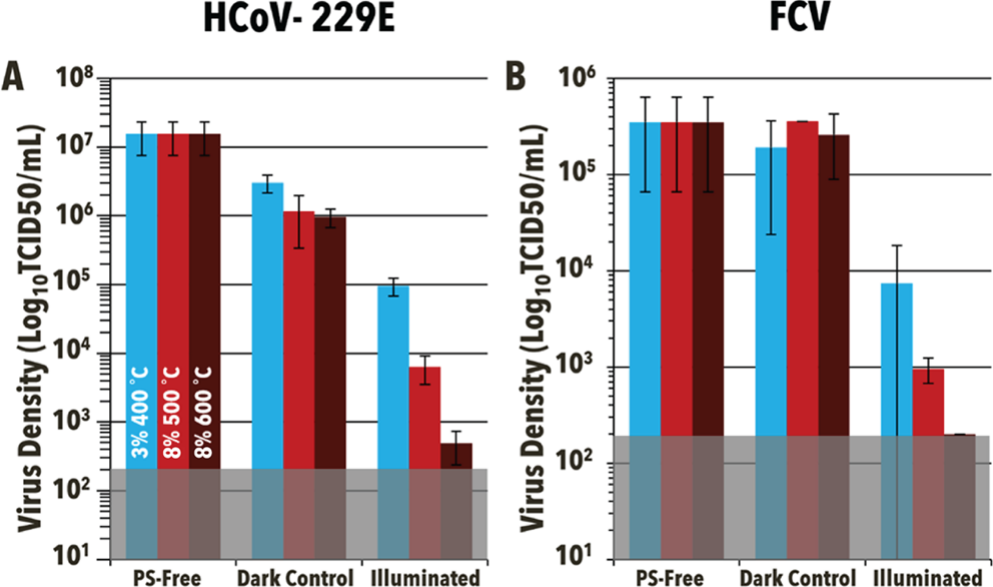
Solution-phase antiviral photodynamic inactivation of (A) Human Coronavirus 229E (HCoV-229E) and (B) feline calicivirus (FCV) using 3%–400 °C, 8%–500 °C, and 8%–600 °C (blue, light red, and dark red, respectively). Both dark controls (Dark) and light reactions (Illuminated, 65 ± 5 mW/cm^2^, 60 min, 400–700 nm) were at particle concentrations of 10 mg/mL and compared to photosensitizer-free controls (PSF) when determining percent survival. The shaded region represents the limit of detection. Error bars correspond to standard deviation.

Solution-based aPDI was performed against multiple viral pathogens to help better understand the particles’ antimicrobial capabilities over a broad range of pathogens. Due to the requirement of biosafety level 3 containment required for SARS-CoV-2, human coronavirus 229E (HCoV-229E), a common cold coronavirus, was used as a reliable surrogate and prime example of an enveloped virus (Figure 9). Even with more gentle illumination conditions (60 min, 65 ± 5 mW/cm^2^, 400–700 nm), the various particles still demonstrated sufficient antiviral capabilities at loading levels of 10 mg/mL, with 99.3% (∼1.7 log units, *p* = 0.0284), 99.95% (∼3.5 log units, *p* = 0.0279), and 99.996% (∼4.6 log units, *p* = 0.0279) for 3%–400 °C, 8%–500 °C, and 8%–600 °C, respectively. To further extend the scope of this study, the particles’ efficacy was also tested against feline calicivirus, a nonenveloped virus. Just as against HCoV-229E, MDRAB, and KP, 8%–600 °C demonstrated the best antimicrobial efficacy inactivating 99.94% (∼3.4 log units, *p* = 0.0005) when compared to 3%–400 °C and 8%–500 °C (97.8%, ∼1.7 log units, *p* = 0.0329 and 99.7%, ∼2.7 log units, *p* = 0.0015, respectively). Similar trends between nonenveloped viruses and higher tolerance to aPDI have been observed in previous studies making the results unsurprising. It is currently hypothesized that nonenveloped viruses have this higher tolerance due to the lack of lipids on the virus surface.^65^

Overall, the concentration of these novel photosensitizers, light irradiance, and time required for inactivation is greater than most previously studied PS, especially water-soluble PS such as methylene blue and rose bengal.^66−68^ While initially concerning, when considering the apparent differences between the photosensitizers it is unsurprising. Perhaps the most important factor is the water solubility of traditional PS studied for aPDI. This solubility allows for constant contact with the pathogen and potential ingestion of the PS, greatly reducing the diffusion distance required for the generated ROS to interact with the bacteria or viruses. To offset this issue, the powders were stirred continuously during experiments; however, contact between particles and pathogens is still expected to be much less than that of a soluble PS.

**Figure 10:**
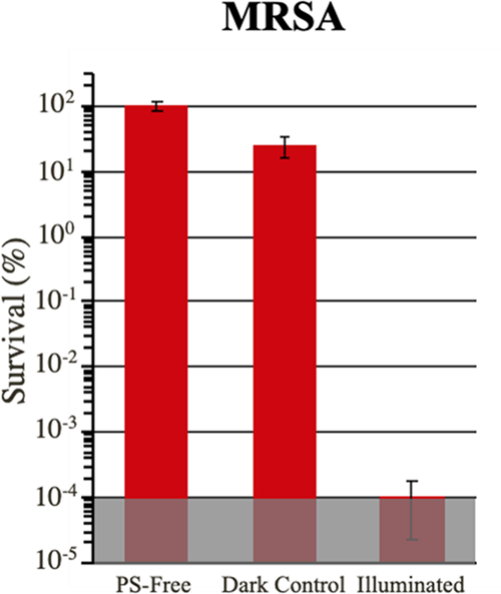
Antibacterial photodynamic inactivation of MRSA using 8%–500 °C after 450 min of exposure to experimental conditions (i.e., five 90 min cycles). Experimental conditions are identical to Figure 7. The shaded region represents the limit of detection. Error bars correspond to standard deviation.

While water-soluble PS have slightly higher efficacies, they suffer from the drawback of photobleaching. When exposed to light for as little as 5 min, the ROS produced by the PS can also attack and degrade the PS itself.^35^ This is a major problem for water-soluble PS as it prevents reuse, and, by extension, represents one of the primary advantages of utilizing solid microparticle PS like those presented in this study. To confirm the overall robustness of our particles, 8%–500 °C powder was stirred in solution under experimental conditions (85 ± 5 mW/cm^2^, 400–700 nm, 90 min) for a total of 4 illumination periods. Between each illumination period, the powder was centrifuged, the supernatant was discarded, and the powder was resuspended in PBS. After the 4 cycles of irradiation and washing, the powder was then exposed to a bacterial solution containing MRSA and irradiated to determine the antimicrobial efficacy after 450 min of illumination (Figure 10). Even after extreme light exposure, the powder still inactivated MRSA to the limit of detection (99.9999%, 6 log units, *p* = 0.0002), thus demonstrating the durability and robustness of the particles. Previous studies investigating copper nanoparticles observed the leaching of copper ions.^17^ To confirm our particles did not have this issue, the collected supernatant was then analyzed via ICP-OES to measure the concentration of copper ions. Copper ion levels ranged between 2.5 and 5 μM, 100-fold lower than what is required for pathogen inactivation as determined through cytotoxicity studies (Figure S8). These results make the application of these novel photosensitizers even more promising, as traditional photosensitizers such as methylene blue commonly suffer from photobleaching after illumination under less intense conditions.

### Preparation and Characterization of Antimicrobial Coatings

**Figure 11:**
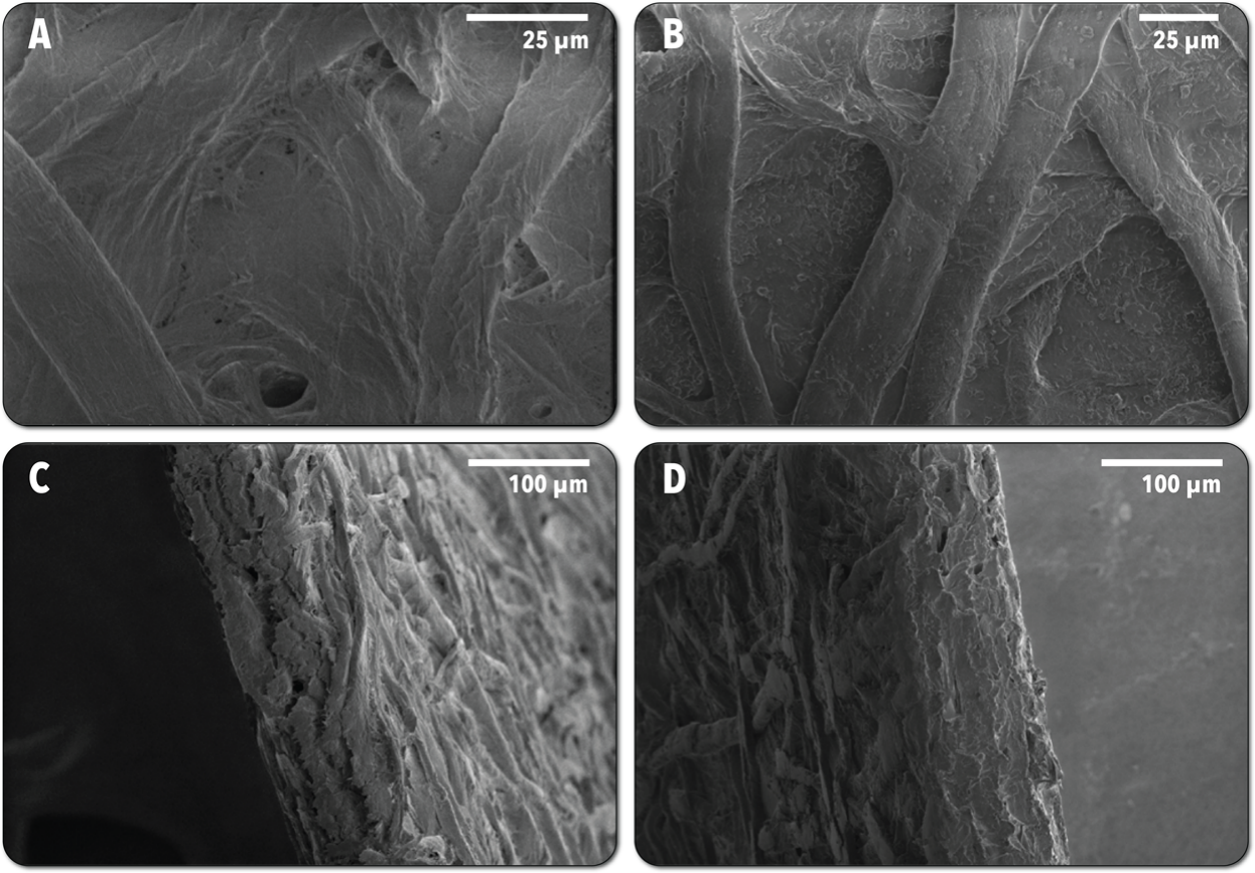
SEM images of coated and uncoated filter paper. (A) Top-down without spray-coat. (B) Top-down with spray-coat. (C) Cross section without spray-coat. (D) Cross section with spray-coat.

After conducting solution-phase aPDI assays using the synthesized particles, a single composition among the best-performing candidates, 8%–500 °C, was selected for incorporation into a polymer coating for application to surfaces. The synthesized powder was ball-milled in water containing BYK as a dispersant and freeze-dried to break apart larger aggregates and create a more homogeneous coating mixture. SEM images of the as-synthesized and ball-milled material can be found in the (Figure S9). Samples were prepared by coating filter paper 3 times with 1.5 mL of 10 mg/mL solution containing 8%–500 °C in SBQ-PVA. A fourth coating of TiO_2_-free SBQ-PVA was then added to act as a final sealant coat. The successful application of the coating was confirmed via SEM shown below in Figure 11. The coated samples are shown from the surface and cross section, with the polymer coating itself more prevalent in the cross-sectional view. The morphology of the substrate remains similar after the coating, indicating that the spray-coated material infiltrates the paper, conformally coating the fibers. The surface image depicts TiO_2_ particles scattered across the surface of the paper, and the cross section further demonstrates the suspected infiltration of the coating polymer as individual fibers are less distinguishable and there is a potential filling of voids in the bulk. This is attributed to the time between coating and curing as the polymer remains fluid until exposed to UV light for approximately 30 min for each coating step. The presence of the Cu-TiO_2_ particles in the coating was confirmed using ICP-OES, where the average loadings were found to be 1407, and 14,083 μg/g of sample for Cu and Ti, respectively, corresponding to an average 0.075 Cu/Ti molar ratio (nominal loading 0.08).

### Antimicrobial Testing of Coated Materials

**Figure 12:**
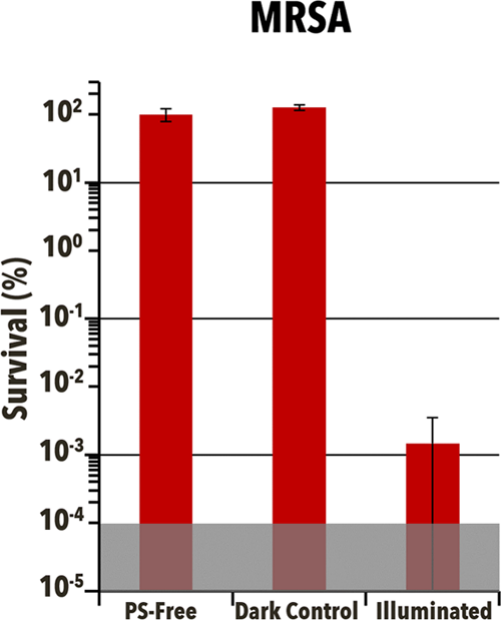
Antibacterial photodynamic inactivation of MRSA on Whatman filter paper spray-coated with 8%–500 °C:SbQ-PVA mixture. Experimental conditions are identical to Figure 7 (illuminated for 90 min at 85 ± 5 mW/cm^2^, 400–700 nm). Error bars correspond to standard deviation.

In a similar fashion to the particles in the solution-based studies, the antibacterial efficacy of the Cu-TiO_2_-coated filter paper was tested against MRSA (Figure 12), exhibiting inactivation up to 99.998% (∼4.8 log units, *p* = 0.003). The lower overall inactivation of pathogens compared to suspended particles can be explained due to the lower overall accessible surface area of particles in the coating. The efficacy of the coated material against MRSA is comparable to textiles that were coated in a similar fashion utilizing water-soluble photosensitizers (97–99.990%).^34^ Previously studied photodynamic materials and coatings^17,66,67^ have shown higher levels of inactivation, yet they required complex synthetic procedures, whereas the current coating procedure is facile, cheap, and easily adaptable.

## Conclusions

In conclusion, we demonstrated a facile synthetic method for preparing light-active copper-doped TiO_2_ microparticles for both antiviral and antibacterial applications. A variety of particle compositions with annealing temperatures ranging from 400 to 600 °C were prepared and characterized. The modified TiO_2_ exhibits increased visible light absorption in the 400–700 nm range relative to undoped samples. The synthesized materials were evaluated for efficacy in solution-phase antimicrobial photodynamic inactivation assays against methicillin-resistant *S. aureus* to identify the best material candidates. Three of the best candidates (99.9999% inactivation), 3%–400 °C, 8%–500 °C, and 8%–600 °C, were then tested against a wide range of other pathogens representing the different categories of bacteria and viruses, including multidrug-resistant *A. baumannii* (Gram-negative), *K. pneumoniae* (Gram-negative drug-resistant variant), feline calicivirus (large nonenveloped), and HCoV-229E (enveloped), with maximal inactivation of 99.9967% (∼5.6 log units), 99.93% (∼3.3 log units), 99.94% (∼3.4 log units), and 99.996% (∼4.6 log units), respectively. The materials were not susceptible to photobleaching after prolonged exposure to ambient light. Furthermore, the samples did not leach sufficient quantities of Cu to result in bacterial inactivation from dissolved Cu ions, confirming that photodynamic inactivation from the Cu-doped TiO_2_ was solely responsible for the inactivation. Finally, a single-material candidate, 8%–500 °C, was incorporated into a polymeric coating, which was tested against methicillin-resistant *S. aureus* where it exhibited 99.998% (4.8 log units) inactivation, effectively demonstrating the applicability of the prepared materials and coatings for use in self-cleaning surfaces and PPE. When compared to previous studies of Cu-doped TiO_2_, the results demonstrated here clearly show their promise in combatting both viral and drug-resistant bacterial pathogen transmission.

## Supporting Information Available

Diffuse reflectance UV–vis spectra of lower Cu compositions; DTA-TGA; additional XRD spectra; STEM-EDS for 3%–400 °C particles; aPDI using P25; aPDI material screening; Cu concentration effects on MRSA inactivation; and SEM of as-synthesized vs ball-milled particles (PDF)
