# Supplementary material for: Facile Synthesis of Cu-Doped TiO2 Particles for Accelerated Visible Light-Driven Antiviral and Antibacterial Inactivation
Source: ACS Appl Eng Mater. 2024 May 3;2(5):1411–23. doi: 10.1021/acsaenm.4c00176 (PMC11129180; doi:10.1021/acsaenm.4c00176)
Supplement: Supplementary file 1 — em4c00176_si_001.pdf [file em4c00176_si_001.pdf]

SUPPORTING INFORMATION

Facile Synthesis of Cu-doped TiO_2 Particles for Accelerated Visible Light-Driven Antiviral and Antibacterial Inactivation

*Zachary S. Campbell,[1,†] C. Roland Ghareeb,[3, †] Steven Baro,[2 , †] Jacob Mauthe,[2] Gail McColgan,[2] Aram Amassian,[2] Frank Scholle, Reza Ghiladi,[3] Milad Abolhasani,[1] Elizabeth Dickey[2,5]**

[1]Department of Chemical and Biomolecular Engineering, North Carolina State University, 911 Partners Way, Raleigh, NC USA 27603

[2]Department of Materials Science and Engineering, North Carolina State University, 911 Partners Way, Raleigh, NC USA 27603

[3]Department of Chemistry, North Carolina State University, 2620 Yarbrough Drive, Raleigh NC, USA 27695-8204

[4]Department of Biological Sciences, North Carolina State University, 3510 Thomas Hall, Campus Box 7614, Raleigh, NC 27695

[5]Department of Materials Science and Engineering, Carnegie Mellon University, 5000 Forbes Ave, Pittsburgh, PA 15213

Email: ecdickey@cmu.edu

Webpage: https://engineering.cmu.edu/directory/bios/dickey-elizabeth.html

S1. UV-Vis Spectra of Lower Cu Composition Materials

In addition to the 3-10% copper materials synthesized in this work, particles containing lower copper loadings (*i.e.*, 1%, 2%) were also synthesized. These loadings were found to cause a red shift in the absorbance of the materials towards the visible range; however, the observed shift in absorbance was viewed as insufficient for the purposes of this work. As such, higher Cu loadings were utilized to influence a greater shift into the visible spectrum. For example, **Figure S1** presents the UV-Vis spectrum for a 2% Cu sample annealed at 500 °C, where there is a small increase in absorbance up to wavelengths of ~500 nm, while materials synthesized with 3% Cu were able to absorb light up to ~600 nm.

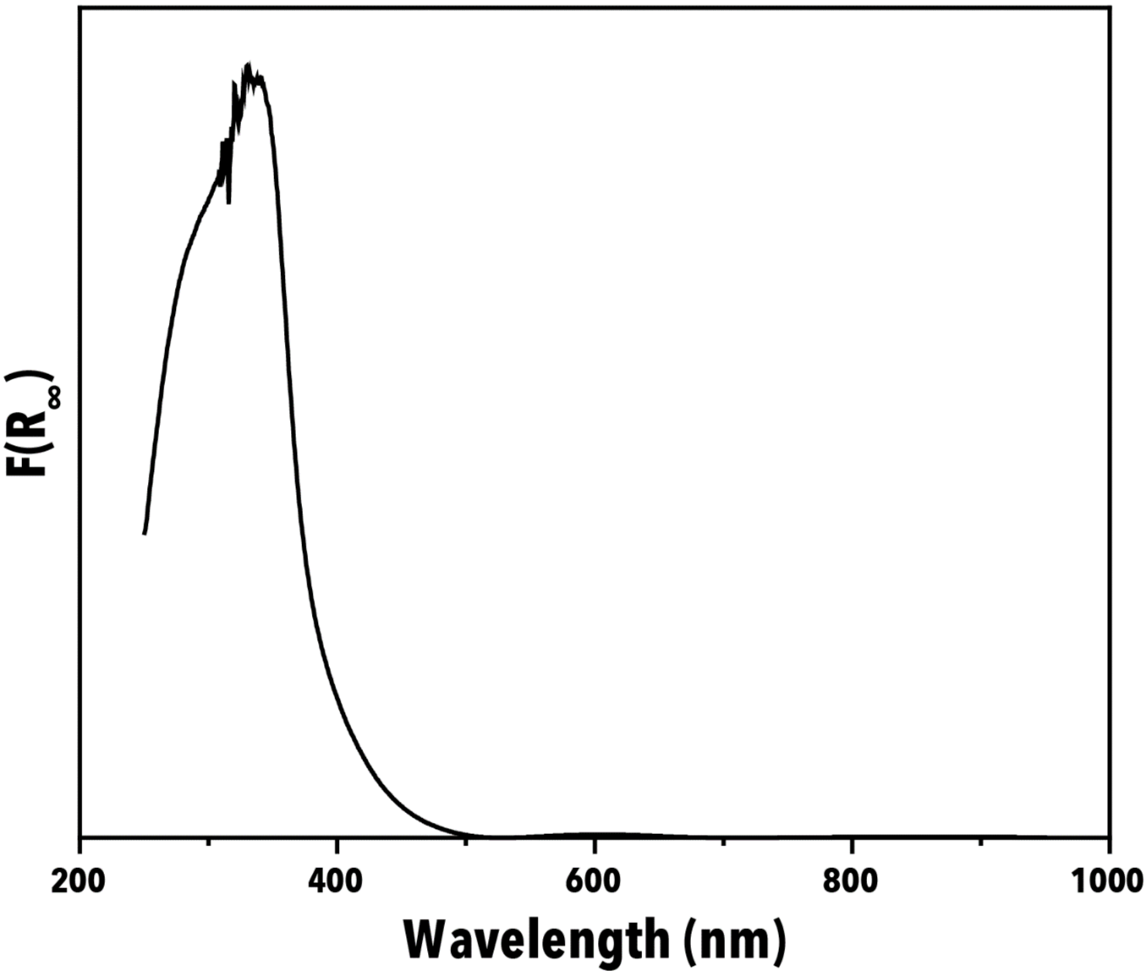

Figure S1. *UV-Vis spectrum for 2% Cu-*TiO_2 *annealed at 500 °C.*

S2. DTA-TGA

The experimental yield of the powder prior to heat treatment was approximately double the theoretical yield by mass, upon heating we observed a range of approximately 40-65% mass loss. This led to the belief that the observed loss is from the removal of residual organics that were incorporated during the synthesis. The thermal and gravimetric profiles were analyzed using DTA-TGA (**Figure S2**). The observed 64% mass loss is in good agreement with a reasonable yield from the synthesis process described previously. The DTA peaks indicate an exothermic reaction that is assumed to be the autoxidation of the aforementioned organics, with the primary reaction onset temperature observed to be near 215 °C, and a smaller exothermic reaction observed to have an onset temperature near 280 °C. Exotherm maximas raised sample temperature to 306 °C and 295.5 °C respectively. The DTA-TGA program mimicked the conditions used for the annealing furnace with a 5 °C/min ramp rate up to 500 °C at which point the sample was held at temperature for an hour.

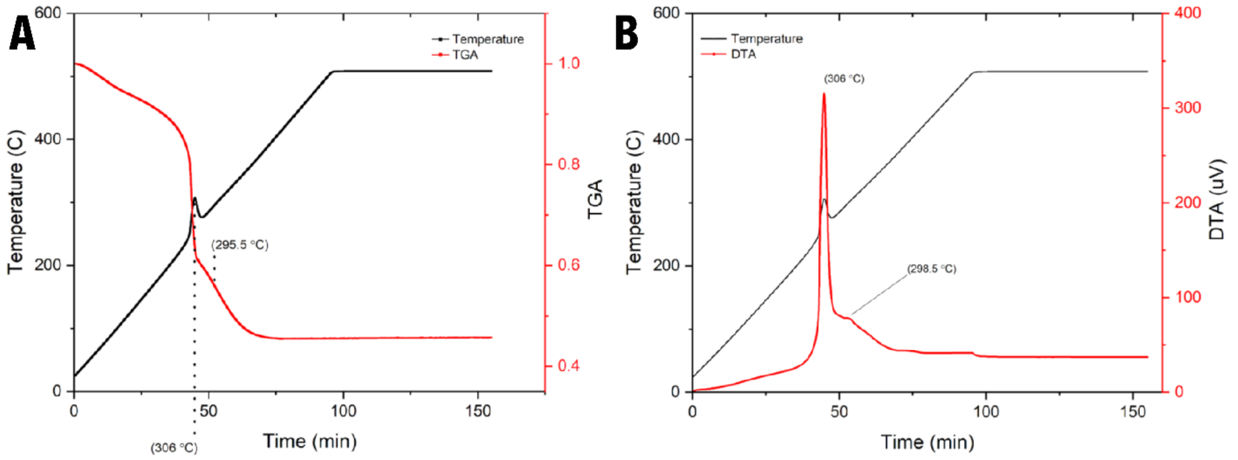

Figure S2. *DTA and TGA data for 8-500 synthesized material. A) Temperature and TGA curves. B) Temperature and DTA curves.*

S3. XRD Spectra

All synthesized samples utilized in this work were characterized via XRD to determine the crystal structure of the particles. **Figure S3** shows the XRD spectra of the compositions not presented in **Figure 2**. It was found that samples annealed at ≤500°C possessed an anatase crystal structure, with the formation of rutile TiO_2 only observed at annealing temperatures of 600°C. Furthermore, at higher Cu loadings (*i.e.*, 8%, 10%), measurable quantities of crystalline CuO were only observed at 600°C annealing temperatures.

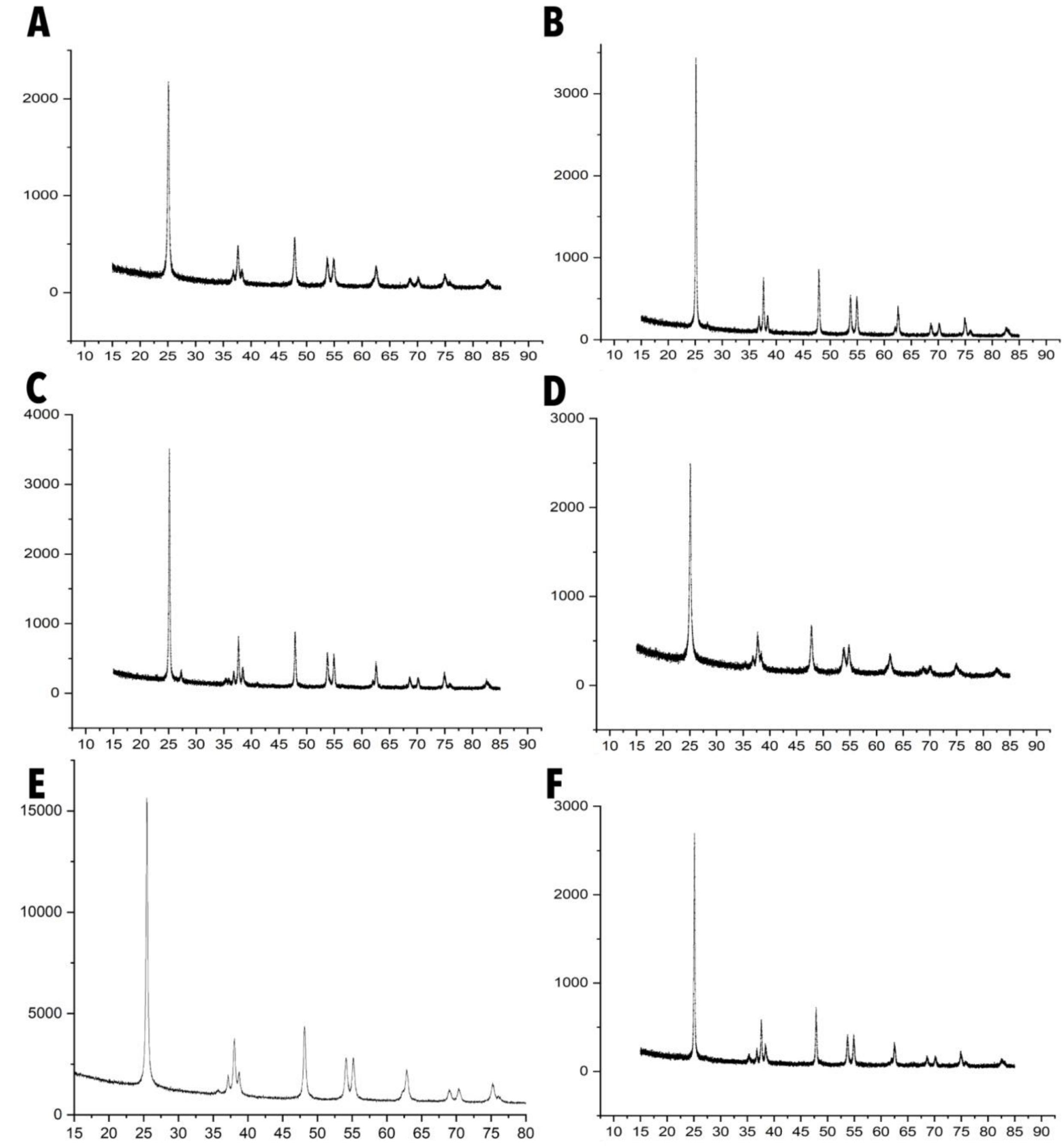

Figure S3. *XRD spectra for the compositions not shown in* ***Figure 2****. A) 3-500, B) 3-600, C) 8-600, D) 10-400, E) 10-500, F) 10-600.*

S4. 3-400 STEM-EDS Data

The synthesized 3-400 particles were also characterized using STEM-EDS. As seen in **Figure S4B-C**, Ti, O, and Cu atoms were well-distributed throughout the particles, which indicates homogeneous Cu doping in the particles. Furthermore, **Figure S4E** shows the C EDS map, where it can be seen that very little carbon is present in the particles, which shows that most of the organics utilized in the synthesis are effectively combusted during the annealing step.

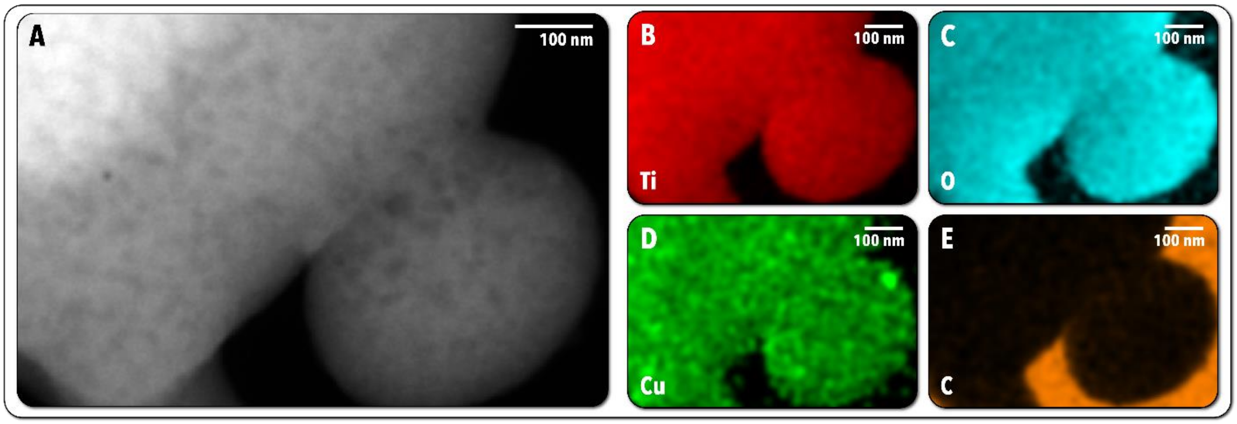

Figure S4*. TEM-EDS data for 3-400 particles. A) HAADF image of the particles. B) Ti EDS map. C) O EDS map. D) Cu EDS map. E) C EDS map.*

S5. P25 Antimicrobial Photodynamic Inactivation

P25 is a readily-available commercial TiO_2 powder that is frequently utilized for photocatalytic applications, it was tested for visible light antimicrobial photodynamic inactivation as a benchmark for the materials synthesized in this work. **Figure S5** shows the inactivation of methicillin-resistant *Staphylococcus aureus* (MRSA) when exposed to P25 with visible light illumination as well as P25-free and dark controls. P25 failed to significantly inactivate MRSA, particularly when compared to the Cu-doped TiO_2 particles synthesized as part of this work.

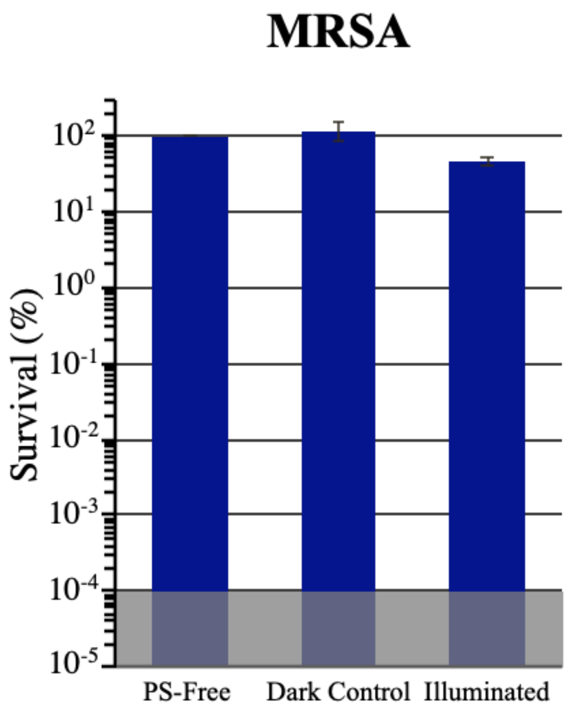

Figure S5. *Antibacterial photodynamic inactivation (aPDI) of methicillin-resistant Staphylococcus aureus (MRSA) using commercially obtained **DP25** at 10 mg/mL. Both coated dark controls (Dark) and light reactions (Illuminated, illuminated for 90 minutes at 85 +/- 5 mW/cm^2) were compared to photosensitizer-free controls (PSF) when determining percent survival. Shaded region represents the limit of detection. Error bars represent standard deviation.*

S6. Material Antimicrobial Photodynamic Inactivation Screening

To evaluate the library of Cu-doped TiO_2 particles for their efficacy for visible-light enabled photodynamic inactivation, each candidate was utilized for aPDI studies against MRSA at a reduced time period and less intense illumination. **Figure S6** shows the results of the screening experiments, where it was found that 3% Cu-doped TiO_2 annealed at 400°C and 8% Cu-doped TiO_2 annealed at 500°C and 600°C were the three best candidates. These compositions were selected for continued use in a wider variety of aPDI experiments.

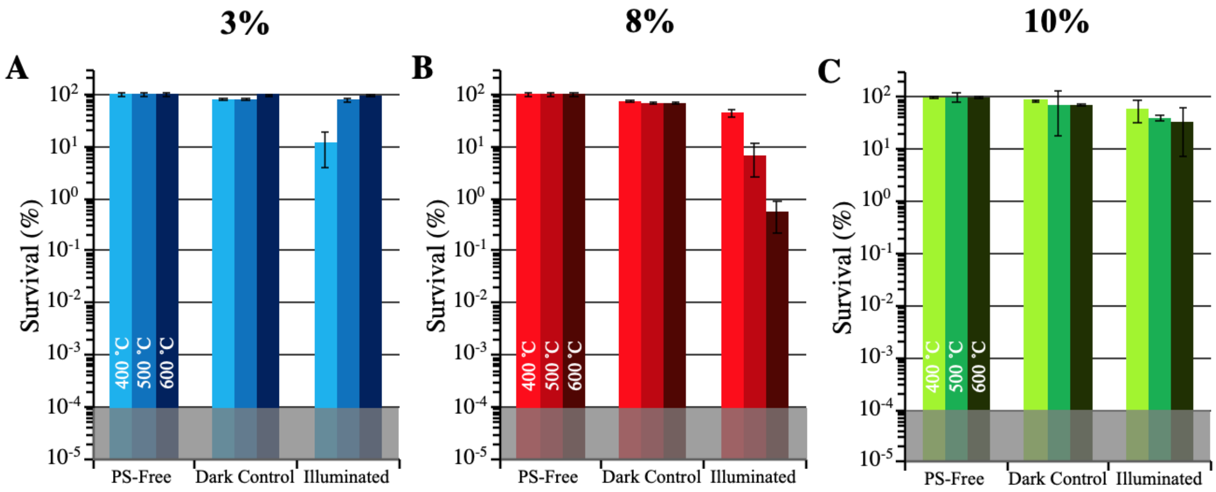

Figure S6. *Antibacterial photodynamic inactivation (aPDI) of methicillin-resistant Staphylococcus aureus (MRSA) using TiO_2 powders annealed at 400, 500, and 600 ˚C and doped with 3% (A), 8% (B), and 10% (C) Cu at 5 mg/mL powder concentrations. Both coated dark controls (Dark) and light reactions (Illuminated, illuminated for 30 minutes at 65 +/- 5 mW/cm^2) were compared to photosensitizer-free controls (PSF) when determining percent survival. Shaded region represents the limit of detection.*

S7. Copper Ion Antimicrobial Inactivation

While the synthesized particles retain most of their incorporated copper, there was still a measurable (if small) concentration of copper that was leached into solution during aPDI experiments. As copper can be cytotoxic to pathogens, tests were completed to explore the effect of copper concentration on MRSA inactivation. As can be seen in **Figure S7**, MRSA was not substantially inactivated until exposed to Cu concentrations of O(100 µM) to O(1000 µM), which are at least 2 orders of magnitude higher than the concentrations reached in solution during the aPDI experiments.

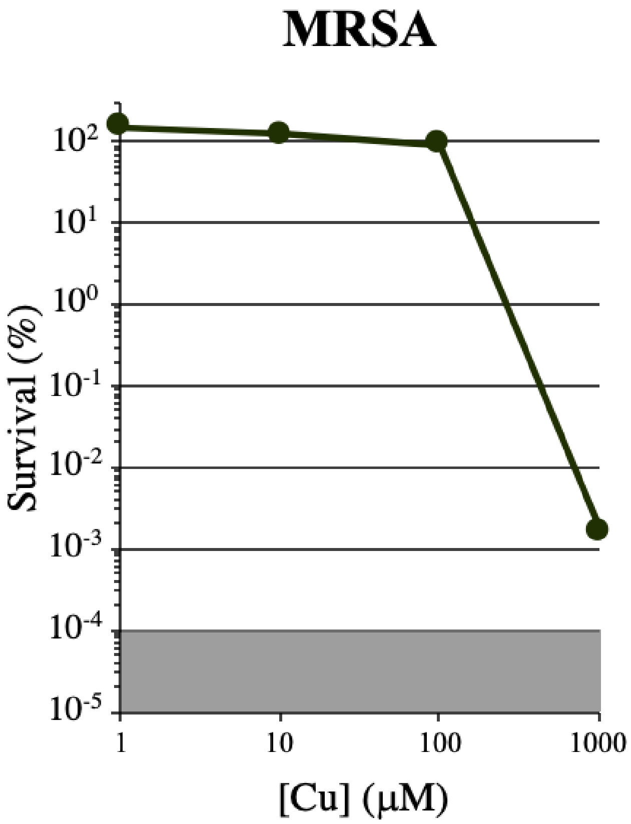

Figure S7. *Antimicrobial efficacy of Cu on MRSA at varying concentrations. Bacteria/copper solutions were stirred for 90 minutes. Cu-containing titers were compared to a control to determine percent survival. Shaded region represents the limit of detection.*

S8. As-synthesized vs. Ball-milled SEM Images

The synthesized particles were also incorporated into spray-coated antimicrobial coatings to explore their efficacy in a more practical environment, specifically to see if they can feasibly be incorporated into personal protective equipment or applied to frequently-contacted surfaces. Prior to coating, it was necessary to ball mill and freeze dry the as-synthesized material, as the particles had a tendency to aggregate during the annealing step. **Figure S8** shows SEM images of both as-synthesized (**Figure S8A, S8C**) and ball-milled material (**Figure S8B, S8D**). It can be clearly seen

that the ball-milled material is much finer, and thus much more easily dispersed in solution for spray coating.

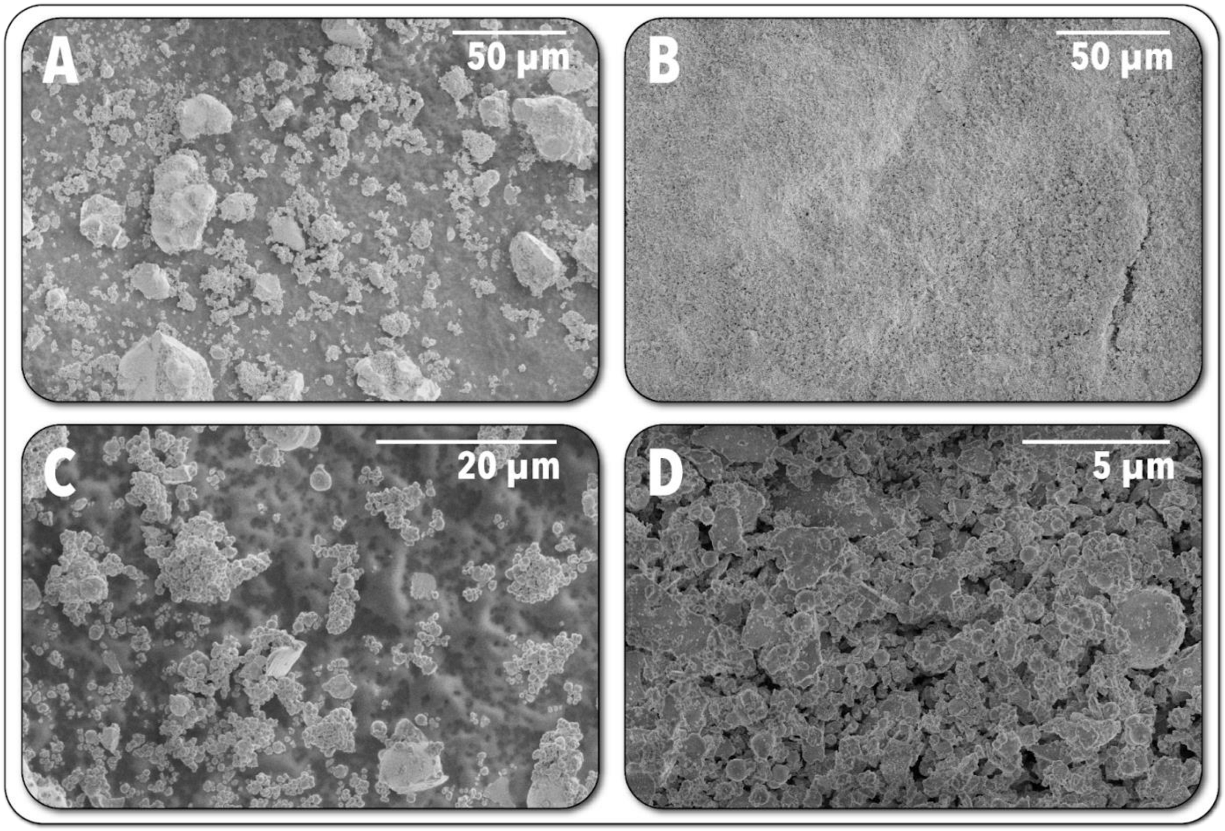

Figure S8. *SEM images of as-synthesized vs. ball-milled 8-500 particles. A) Large field SEM image of as-synthesized particles. B) Large field SEM image of ball-milled particles. C) Higher magnification SEM image of as-synthesized particles. D) Higher magnification SEM image of ball-milled particles.*
